# Case Report: Paradoxical psoriasis under TNF-α blockade may represent generalized abscessing staphyloderma – GASD syndrome by TNF antagonists

**DOI:** 10.3389/fimmu.2026.1726457

**Published:** 2026-06-09

**Authors:** Jörg C. Prinz, Pia-Charlotte Stadler

**Affiliations:** Department of Dermatology and Allergy, University Hospital, Ludwig-Maximilian-University of Munich, Munich, Germany

**Keywords:** adverse event, case report, paradoxical psoriasis, *Staphylococcus aureus*, staphyloderma, TNF-α antagonists

## Abstract

Patients treated with TNF-α antagonists may develop psoriasis-like skin lesions. Clinical manifestations include palmoplantar, inverse, localized or generalized guttate, plaque-type, eczematous, and pustular lesions; deep inflammatory nodes; and nonscarring alopecia, often with overlapping reaction patterns. They are classified as paradoxical psoriasis and interpreted as an inflammatory reaction resulting from the disturbed maturation of plasmacytoid dendritic cells, with overproduction of type I interferons due to tumor necrosis factor α (TNF-α) inhibition. Management frequently necessitates discontinuation of therapy. Here, we review the clinical presentation of six patients who were initially diagnosed with paradoxical psoriasis during TNF-α blockade due to the development of corresponding skin lesions. Dermatologic examination revealed that the lesions were impetigo contagiosa, ecthyma, folliculitis decalvans, ostiofolliculitis, nummular microbial eczema, or abscessing furunculosis—all caused by *Staphylococcus aureus*, likely spreading from the colonized nasal mucosa to the skin. The cutaneous symptoms resolved with topical antimicrobial therapy and systemic antibiotic treatment. We conclude that many cases diagnosed as paradoxical psoriasis may, in fact, represent generalized abscessing staphyloderma resulting from impaired antimicrobial immunity under TNF-α blockade. The diagnosis of paradoxical psoriasis should therefore be critically re-evaluated on a case-by-case basis. Identification and eradication of *S. aureus* may substantially improve the prognosis for patients experiencing paradoxical psoriasis-like manifestations during treatment with TNF-α antagonists.

## 1 Introduction

Tumor necrosis factor α (TNF-α) antagonists are highly effective and indispensable disease-modifying antirheumatic drugs for the treatment of several chronic immune-mediated inflammatory diseases. Approved indications include various forms of arthritis, inflammatory bowel disease, psoriasis, and hidradenitis suppurativa in adults, adolescents, and children (1).

Adverse events associated with TNF-α antagonists primarily arise from their mechanism of action (2). Given the critical role of TNF-α in antimicrobial immunity, patients receiving these agents face an increased risk of bacterial infections (3–6). Furthermore, TNF-α antagonists can induce adverse events that are the opposite of the intended therapeutic effect and are therefore regarded as paradoxical reactions (7). A common adverse reaction during TNF-α blockade is the development of skin changes referred to as paradoxical psoriasis (PDP). PDP comprises a morphologically and histopathologically heterogeneous spectrum of skin alterations (7–12). It includes psoriasiform or eczematous erythematous, often pustular skin lesions; inflammatory nodules or nodes that commonly occur on palms, buttocks, intertriginous groins and axillae, extremities, and trunk; as well as nonscarring, often crusted alopecia lesions on the scalp. Several phenotypes often coexist in the same patient. PDP develops across all indications for TNF-α blockade, including rheumatoid arthritis, ankylosing spondylitis, inflammatory bowel disease, psoriasis, and hidradenitis suppurativa (13, 14). It has been observed with all TNF antagonists, including infliximab, adalimumab, certolizumab, golimumab, and etanercept, with the highest frequency reported during infliximab treatment (9, 11). Paradoxical psoriasis associated with TNF-α antagonists typically occurs within the first 1.5 years of treatment, with a median time to onset ranging between 12 and 14.5 months. However, onset may occur as early as 1 week or as late as several years after initiation of therapy (12, 15, 16). PDP-like episodes have also been reported under immunosuppressive or immunomodulatory treatment with agents other than TNF-α antagonists, e.g., interleukin (IL)-17, IL-12/IL-23, or IL-23 antibodies, as well as Janus kinase (JAK) inhibitors (9). Severe manifestations of PDP may necessitate discontinuation of therapy, depriving patients of a key treatment for managing their debilitating disease (12, 17). With an incidence of up to 10% or higher among treated patients, PDP represents a significant challenge to the long-term use of TNF-α antagonists.

As a proposed pathomechanism of PDP, TNF-α blockade is thought to impair the maturation of plasmacytoid dendritic cells (pDCs), thereby triggering excessive production of type I interferons (IFN-α/β). This interferon overproduction mediates an inflammatory response characterized by a type I interferon signature (18, 19). The underlying mechanisms of PDP induced by other drugs remain unclear, although the clinical phenotype appears similar to PDP associated with TNF-α blockade (9, 14).

Here, we review the clinical presentation and microbial findings of six patients treated with TNF-α antagonists who were referred from rheumatology, gastroenterology, or pediatrics with a diagnosis of PDP (**Table 1**). Our findings indicate that what had been diagnosed as PDP in these patients was, in fact, generalized abscessing staphyloderma (GASD) arising from nasal colonization with *Staphylococcus aureus* due to impaired antimicrobial immunity.

**Table 1:** Skin manifestations, bacteriology, and dermatologic diagnoses of skin lesions in patients with suspected paradoxical psoriasis.

No.	Sex	Age (years)	Underlying disease	TNF antagonist	Major skin changes/type of efflorescence	Main localisation/distribution	Dermatologic diagnosis	Figure No.
1	F	19	AS, Si	Ada, Certo	Sharply demarcated, erythematous, scaling, partially encrusted plaques	Upper and lower extremities	Impetigo contagiosa, nummular eczema	Figures 1, 1A, B
					Disseminated, livid, erythematous papules	Trunk, buttocks, extremities	Folliculitis	Figure 1, 1C
2	F	11	CRMO	Ada, Eta	Eczematous plaques	Axillae	Bacterial intertrigo	
					Livid-colored painful nodules	Groins, inner thighs, trunk	Furunculosis	
					Non-scarring alopecia	Scalp	Folliculitis decalvans	**Figure 1**, **2A**
3	F	28	CD	Ada	Disseminated, erythematous plaques with crusts and desquamation	Great folds	Bacterial intertrigo	**Figure 1**, 3A
					Painful, close-standing livid–erythematous nodules and nodes, partly abscessing	Great folds, buttocks	Furunculosis	**Figure 1**, 3B
					Eczematous erythema	Palms	Eczema	**Figure 1**, 3C
					Partly crusted, scaling erythematous plaques	Extremities	Nummular eczema	**Figure 1**, 3D
					Non-scarring alopecia	Scalp	Folliculitis decalvans	**Figure 1**, 3E
4	F	71	RPA	Eta, Ada	Livid, hyperkeratotic, and partly ulcerated, crusted plaques	Elbows	Ecthymata	**Figure 1**, 4A
					Scattered, livid-colored nodules and pustules	Trunk, axillae	Furunculosis	**Figure 1**, 4A
					Macerated erythema	Bilateral submammary	Bacterial intertrigo	**Figure 1**, 4B
5	M	17	CD	IFX	Pustular rash	Trunk	Ostiofolliculitis Bockhart	**Figure 1**, 5A
					Eczematous plaques	Axillae	Bacterial intertrigo	**Figure 1**, 5A
6	F	42	CD	IFX	Livid, painful papules and nodules, partly abscessing	Buttocks, thigh region, axillae	Furunculosis	**Figure 1**, 6A, B
					Pustules	Mons pubis	Folliculitis	**Figure 1**, 6C

F, female; m, male; AS, ankylosing spondylitis; *Si*, sacroileitis; CRMO, chronic recurrent multifocal osteomyelitis; CD, Crohn’s disease; RPA, rheumatoid polyarthritis; Ada, adalimumab; Certo, certolizumab; IFX, infliximab.

## 2 Methods

The patients had been referred to the outpatient clinic of the Department of Dermatology and Allergy at the University Hospital by other medical specialties within the same institution, with an initial diagnosis of PDP. Each patient was examined by at least two dermatologists. Bacteriological culture smears from skin lesions and nasal vestibules were processed at the Department of Medical Microbiology and Hospital Epidemiology of the Max von Pettenkofer Institute, Ludwig Maximilian University of Munich, Munich, Germany. Histopathological analysis of lesional skin samples was performed at the Department of Dermatopathology, Department of Dermatology and Allergy.

## 3 Results

### 3.1 Patients

#### 3.1.1 Case 1

**Figure 1:**
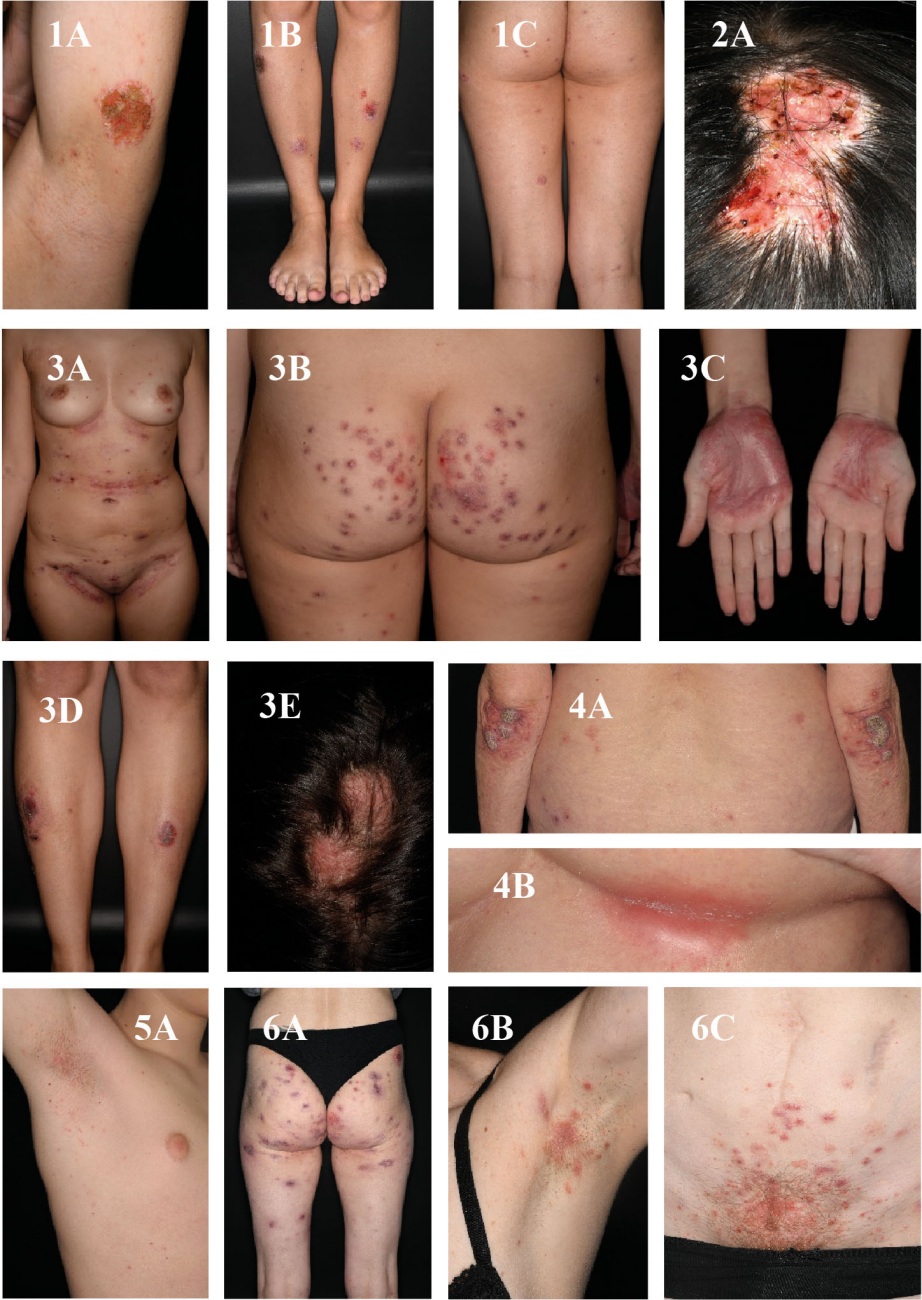
Staphylococci-induced skin lesions that developed under TNF-α blockade, interpreted as paradoxical psoriasis. Numbers and letters refer to the patients and type of skin lesions described in **Table 1**.

Four months after starting treatment, initially with adalimumab and subsequently with certolizumab pegol (**Table 1**), a 19-year-old woman with sacroiliitis and ankylosing spondylitis developed sharply demarcated, erythematous, scaling, and partially encrusted plaques on her upper and lower limbs; disseminated erythematous papules on her trunk and buttocks; and scaling plaques on the scalp (**Figures 1, 1A–C**). Histologic assessment of a lesional biopsy revealed psoriasiform dermatitis with pustule formation. Bacterial swab cultures (BSC) from all sites and the nasal vestibule were highly positive for *S. aureus*. The serum antistaphylolysin antibody titer was 8 IU/ml (< 2 IU/ml). Based on these findings, we revised the diagnosis from PDP to impetigo contagiosa, bacterial folliculitis, and nummular eczema.

#### 3.1.2 Case 2

Six months after starting treatment with adalimumab for chronic recurrent multifocal osteomyelitis (CRMO), an 11-year-old girl developed inflammatory, partially eczematous erythema; multiple deep, livid-colored, painful nodules and pustules, particularly in the groin region, on the inner thighs and axillae, as well as sporadically on the trunk; and nonscarring alopecia with pustules and encrusted erosions on the scalp (**Figure 1**, **2A**). For this reason, treatment was switched from adalimumab to etanercept. As the skin lesions continued to worsen, the TNF-α blockade was eventually discontinued, resulting in the recurrence of CRMO accompanied by severe pain. BSC from the nostrils, axillae, skin, and alopecia lesions consistently detected *S. aureus*. The dermatological diagnosis was bacterial intertrigo, furunculosis, and folliculitis decalvans rather than PDP.

#### 3.1.3 Case 3

During a 13-month course of treatment with adalimumab for Crohn’s disease, a 28-year-old woman gradually developed disseminated erythematous plaques with crusts and scaling; painful, tightly packed livid–erythematous nodes and nodules in the large folds and on the buttocks; eczematous erythema on the palms; and alopecia lesions on the scalp (**Figure 1**, 3A–E). BSC from the skin lesions and nasal vestibules revealed growth of *S. aureus*. Instead of PDP, the dermatological diagnosis was bacterial intertrigo, furunculosis, nummular eczema, and folliculitis decalvans.

#### 3.1.4 Case 4

Two years after starting treatment with etanercept for rheumatoid polyarthritis, a 71-year-old patient progressively developed livid, hyperkeratotic, and partly ulcerated, crusted plaques on both elbows; scattered livid-colored nodules and pustules disseminated over the free integument and in the axillae; and macerated erythema under the breasts (**Figure 1**, 4A, B). The skin lesions continued to worsen after switching to adalimumab. Histological evaluation of two lesional biopsies revealed lichenoid interface dermatitis with neutrophils and eosinophils. BSC from the skin lesions and nasal mucosa were consistently positive for *S. aureus*. The diagnosis was revised from PDP to ecthymata, bacterial intertrigo, and furunculosis.

#### 3.1.5 Case 5

Within 5 months of starting treatment with infliximab for Crohn’s disease, a 17-year-old boy developed a pustular rash on the trunk and eczematous lesions in the axillae, initially diagnosed as PDP (**Figure 1**, 5A). BSC from the lesions, axillae, and nasal vestibules revealed growth of *S. aureus*. The diagnosis was subsequently revised to Bockhart’s ostiofolliculitis and bacterial intertrigo.

#### 3.1.6 Case 6

During a 4-year course of treatment with infliximab for Crohn’s disease, a 42-year-old woman experienced recurrent bluish, painful papules and nodules, as well as erythematous plaques primarily localized on the buttocks and thighs, and pustules on the mons pubis (**Figure 1**, 6A–C). The lesions worsened further with the additional administration of ciclosporin for suspected PDP. *S. aureus* was cultured from lesions on the mons pubis, buttocks, groin, axillae, nasal vestibules, and pharynx. The dermatological diagnosis was furunculosis and bacterial folliculitis.

### 3.2 Clinical approach to diagnosis

Following the classic rule of dermatological diagnosis, which combines the type and distribution of efflorescences, we diagnosed various forms of staphylococcal skin infection in every patient initially diagnosed with PDP. These included impetigo contagiosa, ecthyma, bacterial intertrigo, folliculitis decalvans (perifolliculitis capitis abscediens et suffodiens), ostiofolliculitis, nummular microbial eczema, and abscessing furunculosis (20, 21) (**Table 1**; **Figure 1**). The diagnoses of staphylococcal skin infections were confirmed by BSC. Dense colonization of the nasal vestibules with *S. aureus* was found in all patients, representing the likely source of bacterial dissemination. In all cases, the isolated strains of *S. aureus* were sensitive to methicillin, i.e., susceptible to beta-lactam antibiotics.

### 3.3 Course of GASD following staphylococcal eradication, illustrated by case 2

**Figure 2:**
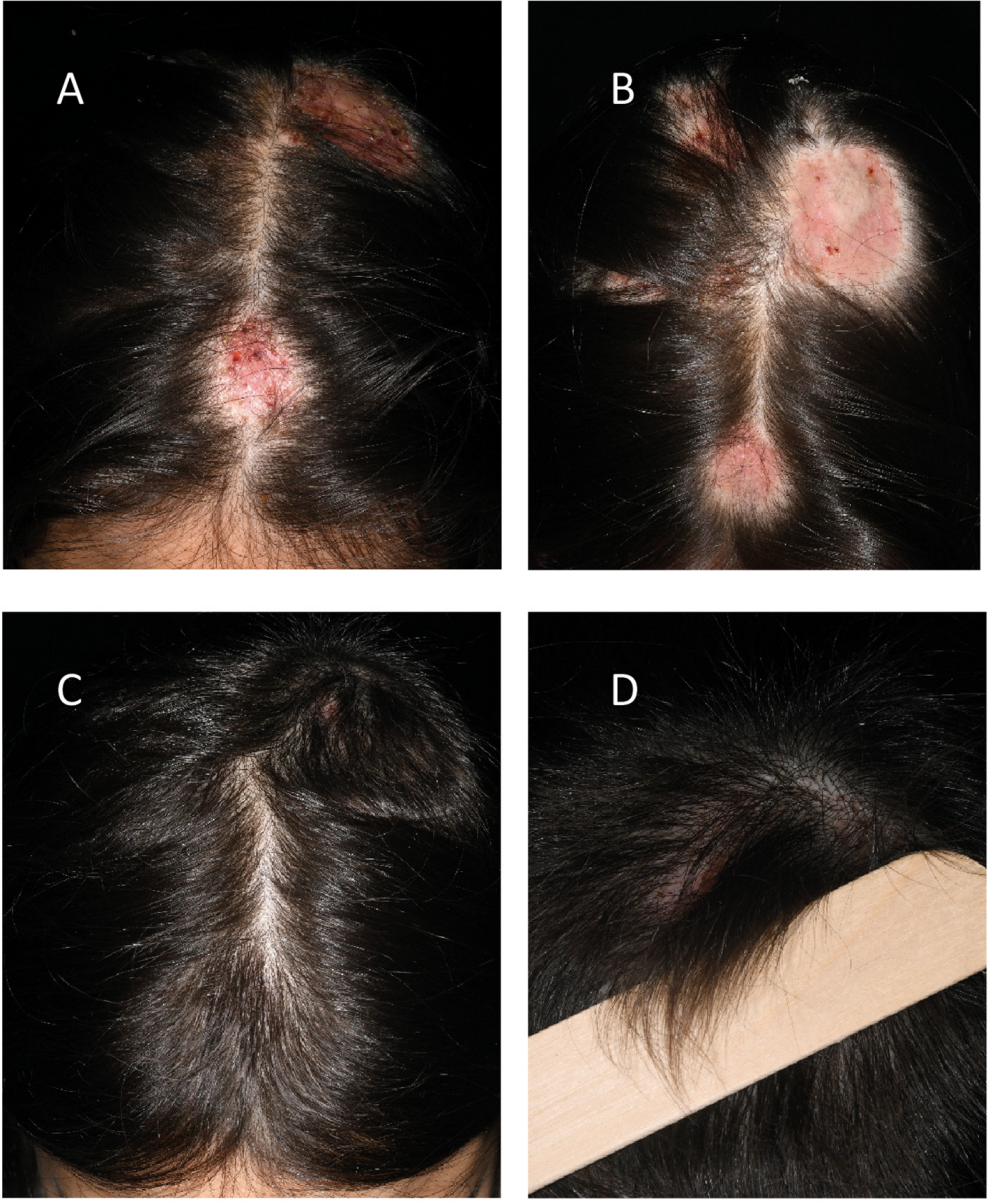
Course of alopecia lesions classified as paradoxical psoriasis of patient 2. Photographs show the lesions at the beginning of antibiotic and topical antimicrobial therapy **(A)**, and at follow-up examinations after 4 **(B)** and 16 weeks **(C)**, with regrowth of hair in the former alopecia lesions despite continued TNF-α blockade **(D)**.

In all patients, the skin lesions regressed following local antimicrobial and systemic antibiotic therapy. Case 2 is representative of the response of PDP-like skin changes to antimicrobial and antibiotic treatment. Following the onset of the skin lesions with adalimumab, treatment was switched to etanercept, which was then discontinued when the condition worsened further, leaving the CRMO untreated despite severe pain. Following the detection of *S. aureus* on all skin lesions and the nasal vestibules, we initiated a combination of systemic antibiotics and topical antimicrobial therapy. It consisted of cefaclor 500mg 3 x daily for 10 days according to the broad antibiotic staphylococcal sensitivity, mupirocin 20mg/g nasal ointment for the nasal mucosa, and polyhexanide wound gel for the skin lesions. Cefaclor was prescribed as it is indicated for uncomplicated infections of the skin and skin structures caused by methicillin-sensitive *S. aureus* strains, and is available as an oral suspension for children who are unable to swallow capsules. In parallel with the antimicrobial therapy, treatment with etanercept was resumed (2 × 50 mg/week). **Figure 2** shows the course of regression of the alopecia lesions presented in **Figure 1**, **2A** from the onset of antimicrobial therapy (**Figure 2A**). On re-examination 4 (**Figure 2B**) and 16 weeks (**Figure 2C**) later, the pustules and inflammation of the alopecia lesions had largely regressed, and the bacterial culture smears from the alopecia lesions, axillae, and nasal vestibules grew normal skin or nasal flora. Further improvement was observed in the weeks that followed, with normal hair regrowth noted by week 16 (**Figure 2D**). Thus, all skin lesions healed with systemic antibiotic and topical antiseptic therapy alone, despite the simultaneous reintroduction of TNF-α blockade. Precise instructions and a high compliance of the patient and her parents with regard to topical antimicrobial therapy and the prevention of further nasal colonization with staphylococci contributed to this outcome.

## 4 Discussion

### 4.1 Interpreting the clinical findings of PDP cases in the context of the literature

The time between the initiation of TNF-α blockade and the onset of the first symptoms varied among our patients, ranging from a few weeks to 2 years. This is consistent with the timeframes reported in the literature for the initial manifestation of PDP (12, 15, 16). In terms of morphology, localization, and histopathological analysis, the patients described here reflect the spectrum of skin lesions observed during TNF-α blockade, being morphologically indistinguishable from those diagnosed as PDP in various reports (9–12, 18, 22, 23). Although this small series of cases does not allow for reliable conclusions about the general gender distribution in PDP, the predominance of female patients is nevertheless consistent with the findings from systematic reviews and meta-analyses. These findings indicate that female patients undergoing treatment with TNF inhibitors have a significantly higher risk of developing PDP, with the female-to-male ratio reaching as high as 3:1 depending on the population studied (13, 24, 25).

According to the particular type and distribution of efflorescences, the six patients exhibited the classic spectrum of skin lesions caused by staphylococcal skin infection. Consistent with this, *S. aureus* was consistently detected in all bacterial swab cultures from skin lesions and the nasal mucosa. High antistaphylolysin (ASTA) serum antibody titers determined in case 1 further indicated extensive staphylococcal exposure. Skin lesions regressed with systemic antibiotics in combination with topical antimicrobial therapy, supporting that *S. aureus* was indeed the causative agent rather than a secondary colonizer, as previously suspected (10). Thus, in all six patients, the skin lesions that developed under TNF-α blockade were classified as GASD.

Nasal carriage of *S. aureus* with a high bacterial load, observed in all patients, is likely to be particularly relevant to the development of the skin changes interpreted as PDP during TNF-α blockade. The nasal mucosa serves as the primary reservoir for *S. aureus*, from which the bacteria can spread to various body sites and cause skin infections (26). Impaired antimicrobial immunity caused by TNF-α blockade or other disease-modifying anti-rheumatic drugs (DMARDs) may promote continuous nasal colonization with *S. aureus* (27). Intertriginous regions are predilection sites for *S. aureus* growth and may explain the “inverse” localization of PDP skin changes in these regions (28).

In their study on the pathogenesis of PDP, Conrad et al. examined various immunological parameters (18). While IL-17A, IL-17F, IL-17C, IL-26, IFNG, IL-4, and IL-10 showed comparable levels in skin biopsies from paradoxical and classical psoriasis, they observed significantly increased expression levels of type I interferons (IFN-α/β) and IL-22 in paradoxical psoriasis lesions. These findings correlated with a marked accumulation of pDCs in the paradoxical skin lesions, suggesting that pDCs represented the actual source of type I interferons. Impaired maturation of pDCs, with increased type I IFN expression in the presence of TNF blockers, was observed when human peripheral blood mononuclear cells were stimulated with LL37/DNA complexes and in a skin injury mouse model. From these observations, the authors concluded that paradoxical psoriasis represents an overactive, type I IFN-driven innate inflammation in which TNF-α antagonists tip the balance towards type I interferons, ultimately driving the inflammatory psoriatic phenotype of PDP.

However, clear parallels between the features of PDP and staphylococcal skin infections (**Table 2**) suggest that the changes attributed to cytokine imbalance from TNF-α blockade may have a different etiology. Skin infection with *S. aureus* robustly activates the innate immune response, as observed in PDP lesions (29). Infection recruits pDCs into the skin (30), which may explain their abundance in PDP lesions. Recognition of staphylococcal components by pDCs via pattern recognition receptors induces abundant production of IFN-α, an effect primarily triggered by staphylococcal protein A through TLR9 ligation (31–37). Staphylococcal toxins induce the expression of IL-22 (38), which is augmented in cells of the dense inflammatory infiltrate in skin lesions diagnosed as PDP (22). IL-22 plays a critical role in the immune response to staphylococcal skin infection by promoting keratinocyte proliferation and inducing antimicrobial peptides (39–43). Mouse and human studies demonstrate that *S. aureus* infection drives Th17/Th22 responses, which are important for bacterial clearance (44–46), explaining why suspected PDP and plaque psoriasis exhibit similar IL-17 expression levels (18, 47, 48). Staphylococcal skin infection induces the expression of IL-36 (49) and rapidly recruits polymorphonuclear leukocytes and eosinophils to the site of infection (29, 50, 51), all of which characterize skin lesions diagnosed as PDP (8, 19, 52). The obvious parallels between staphylococcal skin infections and PDP, with evidence of *S. aureus* in all PDP lesions, thus support the assumption that the changes classified as PDP may in fact be due to skin infection with *S. aureus*. An infectious etiology from impaired anti-microbial immunity would furthermore explain the development of PDP-like skin changes observed during treatment with other biologics or JAK inhibitors (14), as well as the heterogeneous morphology and spectrum of the skin lesions—even within the same patient—reflecting different manifestations of staphylococcal skin infections rather than a uniform pathomechanism based on cytokine imbalance.

**Table 2:** Parallels between the hallmarks of PDP by TNF-α blockade and staphylococcal skin infection.

Characteristic features	PDP	Staphylococcal skin infection
Activation of the innate immune response	Yes (18)	Yes (29)
Accumulation of pDCs	Yes (18)	Yes (30)
Type I interferons	High expression in PDP lesions (18)	Induced by staphylococcal skin infection (31–37)
IL-22	High expression in PDP lesions (18, 48)	Induced by staphylococcal skin infection (38)
Neutrophilic granulocytes	Neutrophils infiltrating the stratum corneum, epidermis, and dermis, with microabscesses (52)	Recruited in staphylococcal skin infection (29, 50)
Eosinophils	Accumulation characteristic of PDP lesions (8, 19, 52)	Recruited in staphylococcal skin infection (51)
IL-17	Expressed in PDP lesions similar to psoriasis (18, 48)	Induced by staphylococcal skin infection (44, 45, 51)
IL-36	Expressed in PDP lesions (18)	Induced by staphylococcal skin infection (29, 47, 49)
Antimicrobial peptides	Expressed in PDP lesions (41)	Induced by staphylococcal skin infection (42, 43)

### 4.2 Practical recommendations in cases of suspected PDP

The clinical morphology of PDP lesions reflecting bacterial skin infections, the detection of *S. aureus* in the paradoxical psoriasis skin lesions—likely disseminated from the nasal vestibules—increased numbers of pDCs, elevated expression of type I interferons and IL-22 in the skin lesions, as well as improvement after antimicrobial treatment in our patients, are all consistent with an *S. aureus*-mediated etiology of PDP. The assumption that PDP may represent staphylococcal skin infection in many patients requires a change in management strategies focusing on *S. aureus*. Bacterial examination is, therefore, an important diagnostic procedure when PDP-like skin changes occur. The nasal vestibules should always be examined for colonization with *S. aureus*, as they represent the likely source of spread. If *S. aureus* is detected, antibacterial eradication therapy can lead to recovery and may prevent the need to change or discontinue treatment with biologics. Recommended measures include:

- Thorough bacterial diagnostics by swab culture from skin lesions and nasal vestibules, and, if possible, determination of serum antibody titers against staphylococcal toxins;
- Sanitation of the nasal mucosa with topical antibiotic or antimicrobial agents (e.g., mupirocin, polyhexanid);
- Systemic antibiotic therapy guided by culture sensitivity (antibiogram);
- Topical antimicrobial therapy for skin lesions (e.g., octenidine dihydrochloride, polyhexanide);
- Strict personal hygiene with antiseptic washes;
- Avoiding manual nose-picking, as *S. aureus* is frequently transmitted between the nasal mucosa and hands (26);
- Ongoing hygiene measures to prevent recurrences of staphylococcal infection.

Overall, the data presented here may explain the development of PDP-like skin lesions through infection with *S. aureus*, facilitated by an impaired antimicrobial immune response. These findings suggest that the current understanding of the pathogenesis and diagnosis of paradoxical psoriasis requires a critical re-evaluation through a systematic, evidence-based approach that integrates clinical observations, microbial findings, and evolving immunological insights to distinguish infectious causes from a dysregulated cytokine response due to TNF-α blockade or other DMARDs. In particular, the role of *S. aureus* in PDP warrants further systematic investigation, as no data or studies specifically addressing this aspect have been reported to date. In addition to BSC, such investigations may involve serological methods measuring antibody titers against staphylococcal toxins, such as the staphylococcal alpha-toxin/alpha-hemolysin (ASTA titer), or other serological tests (53). Elevated ASTA titers, as seen in case 1, reflect a sustained immune response to *S. aureus*, correlating more closely with the duration and depth of tissue involvement rather than merely the presence of infection (54). Overall, such studies should provide further insights into the role of *S. aureus* infections as a potential cause of PDP. Identification and thorough eradication of *S. aureus* as a potential cause of PDP-like symptoms may greatly improve patient outcomes during treatment with TNF-α antagonists or other DMARDs, often without the need to alter or discontinue therapy. Addressing this issue is of great importance, as TNF-α antagonists are used worldwide across numerous indications. The cumulative exposure could reach tens of millions of patients by 2025, although no consolidated figures are currently available.

## Data Availability

The raw data supporting the conclusions of this article will be made available by the authors, without undue reservation.
